# Vitamin D3 levels and NLRP3 expression in murine models of obese asthma: association with asthma outcomes

**DOI:** 10.1590/1414-431X20176841

**Published:** 2017-11-13

**Authors:** J-H. Zhang, Y-P. Chen, X. Yang, C-Q. Li

**Affiliations:** 1 Department of Emergency, the First Affiliated Hospital of Guangxi Medical University, The Guangxi Talent Highland for Emergency and Rescue Medicine, Guangxi Colleges and Universities Key Laboratory of Emergency Medicine Research, Nanning, Guangxi, China; 2 Department of Geriatrics, Guangxi Minzu Hospital, The Affiliated Minzu Hospital of Guangxi Medical University, Nanning, Guangxi, China; 3 Department of Respiratory Medicine, Guangxi Vocational and Technical College of Health, Nanning, Guangxi, China

**Keywords:** Asthma, Obesity, Airway hyperreactivity, NLRP3 inflammasome, Vitamin D3

## Abstract

Vitamin D (25(OH)D3) is an essential nutrient that plays a role in the immune system. Serum 25(OH)D3 is found to be associated with asthma. However, the role of vitamin D in obese asthma remains unclear. Therefore, we investigated the association between vitamin D levels and asthma outcomes in a murine model of obese asthma. We also evaluated NLRP3 inflammasome activity in the pathogenesis of obese asthma. We divided 20 male Balb/c mice (3–4 weeks old) into 4 groups: normal control, asthma, obese, and obese asthma and developed an obese asthma mouse model. Airway hyperreactivity, cytokine concentrations, 25(OH)D3 levels, NLRP3 mRNA and IL-1β mRNA expressions were measured. Lung histology and bronchoalveolar lavage fluid (BALF) cell count were also determined. Obese asthma mice showed a significant increase in airway hyper-responsiveness, airway inflammation, pro-inflammatory cytokine levels and NLRP3 mRNA, IL-1β mRNA expression. Both asthma and obese groups had lower 25(OH)D3 levels. Vitamin D levels in obese asthma were the lowest among all groups. Vitamin D levels correlated negatively with body weight, lung resistance levels at 25 mg/mL of methacholine, total inflammatory cells, and IL-1β and IL-17 concentrations in BALF. These data demonstrated an association between serum vitamin D levels and outcomes of obese asthma, and indicated that NLRP3 inflammasome may play a role in this disorder.

## Introduction

Obesity and asthma are major health problems with a growing worldwide prevalence. In recent decades, both diseases have increased substantially and almost synchronously. Recently, researchers have reported that obese asthmatics may represent a clinically distinct subset of asthma (1). Moreover, obese asthmatics respond poorly to typical asthma medications and their symptoms appear to be more severe (2). However, in spite of the disclosed link between asthma and obesity, the mechanism of their association remains unclear.

Vitamin D deficiency has a relationship with the incidence of asthma, as shown by epidemiological information. Studies indicate that vitamin D has anti-inflammatory effects on chronic lung inflammation and is a potential regulator of the development of respiratory diseases including asthma and chronic obstructive pulmonary disease. On the other hand, low levels of circulating vitamin D3 correlate with high adiposity. Therefore, these findings suggest that vitamin D may play a role in obesity and asthma.

Moreover, NLRP3 inflammasome is also critical in the development of allergic airway inflammation and in the pathophysiology of obesity. This study aimed to measure the expression of NLRP3 mRNA and IL-1β mRNA in lung tissue, and vitamin D levels in serum in a model of obese asthmatic mice. In addition, we evaluated the relationship of serum vitamin D levels and obese asthma outcomes, and the activity of NLRP3 inflammasome in the animals.

## Material and Methods

### Animals

Twenty specific pathogen-free (SPF) BALB/C mice (3–4 weeks old, male) were purchased from Laboratory Animal Center of Guangxi Medical University (Nanning, Guangxi, China). Mice were given food and water *ad libitum*, placed in a 12:12-h light-dark cycle, and fed for 8 weeks with either a standard chow diet (70% carbohydrate; 20% protein, 10% fat) or a high-fat diet that induces obesity (29% carbohydrate, 16% protein, 55% fat). Animal care and experimental protocols followed the Guide for the Care and Use of Laboratory Animals, and experimental protocols were approved by the Committee of Ethical Principles in Animal Research adopted by the Guangxi Medical University for Animal Experimentation.

### Experimental protocols and allergen sensitization

Mice were randomly divided into four experimental groups (5 mice/group): control group (group A), obesity group (group B), asthma group (group C) and obese asthma group (group D). The mice of groups B and D were fed with the high-fat diet to achieve a diet-induced obesity model according to the method of a previous study (2). Groups A and C were fed the standard mouse chow. After 8 weeks of feeding, sensitization and challenge with ovalbumin (OVA) in groups C and D were performed using a method described previously (3). Briefly, 25 µg OVA (grade V; Sigma, USA) and 1 mg Al(OH)3 (Sigma) were injected *ip* on days 1, 7, and 14. Mice were then challenged with aerosol OVA (2% in saline) for 20 min daily from day 21 to day 28. During the experiment, body weight and length were measured weekly.

### Airway responsiveness test

Airway responsiveness was measured using a noninvasive pulmonary function instrument (Fine-Pointe NAM system TBL3999, Buxco, USA). The test procedures were performed as previously described (4). The data are reported as lung resistance (RL, cmH_2_O·s^-1^·mL^-1^) to determine the airway responsiveness of each mice.

### Sample collection

After the last aerosol challenge, the mice were sacrificed with an overdose of pentobarbital sodium (100 mg/kg body weight, *ip* injection). Lung tissue, bronchoalveolar lavage fluid (BALF) and serum samples were harvested. The serum was isolated and stored at −80°C until assay. BALF was obtained using the method described previously (3). The collected fluid was then centrifuged at 500 *g* for 2 min at 4°C. The supernatant was collected and stored at −20°C for cytokine determination. The right lobes were fixed in formalin for lung histology analysis and the left lobes were stored at −80°C for RT-PCR measurement.

### Total cell counts and differential cell counts in BALF

The cell pellet was resuspended by 200 µL PBS. Fifty microliters of cell suspension was measured using a hemocytometer. Differential cell counts were assessed. Another 50-µL suspension was subjected to cytospin at 450 rpm for 5 min, followed by Diff-Quick staining (Sysmex Corporation, Japan) to detect inflammatory cells. A total of 300 cells were counted under microscopic examination. Types of inflammatory cells were determined in randomly selected fields of the slide with a differential cell counter (Hwashin Tech, Korea) based on morphologic criteria and staining characteristics. Inflammatory cells were classified as eosinophils, neutrophils, macrophages, or lymphocytes.

### Lung histologic examination

The lung was fixed in 4% paraformaldehyde in PBS and paraffin-embedded. Lung sections of 4 μm were stained with hematoxylin and eosin (HE) and periodic acid-Schiff (PAS) for morphological evaluation. The inflammatory cells, airway mucosal epithelial cells, the amount of goblet cells and mucus production around the bronchial and blood vessels of the right lung tissue were observed under a light microscope (Olympus, Japan). Lung inflammation was estimated by semi-quantitative evaluation by the peribronchovascular inflammatory cell infiltrations (0, none; 1, mild; 2, moderate; 3, marked; 4, severe) and the proportion of goblet cells in airway epithelial cells (0, none; 1, <25%; 2, 25–50%; 3, 51–75%; 4, >75%).

### Measurement of vitamin D3 in serum

Quantitation of the serum vitamin D3 was performed by ELISA according to the manufacturer's instructions. The ELISA kits were purchased from Cusabio Biotech Co. Ltd (Catalog Numbers: CSB-E08099m, CUSABIO BIOTECH, Wuhan, China). Absorbance of each sample was determined at 450 nm using a micro-plate reader (Bio-Rad Laboratories, Hercules, CA, USA).

### Analysis of cytokine concentrations in BALF

The supernatant of BALF suspension was used for cytokine quantification. Amounts of cytokines IL-1β and IL-18 in BALF were analyzed by ELISA using R&D Systems (USA) following the manufacturer's instructions. The BD™ Cytometric bead array (CBA) mouse Th1/Th2/Th17 kit (BD Biosciences, USA) was used to detect cytokine concentrations of IL-2, IL-4, IL-6, IL-10, IFN-γ, TNF-α and IL-17A, and sample analysis were done using a FACScalibur flow cytometer.

### Real-time RT-PCR

Total RNA was prepared from the lung tissues using Trizol Reagent (Invitrogen, USA), and complementary DNA (cDNA) was synthesized according to manufacturer's instruction using a cDNA reverse transcription kit (Invitrogen). Quantitative PCR analysis was performed with the SYBR qPCR mix (Toyobo, Japan). Quantitative PCR products were measured using an Applied Biosystems 7500 Sequence Detection System (Applied Biosystems, USA) to determine the IL-1β mRNA and NLRP3 mRNA expression. The levels of target genes expression were normalized to β-actin expression using the 2^−ΔΔCt^ method.

The sequences of the forward and reverse primers are as follows: mouse NLRP3, 5′-GACACGAGTCCTGGTGACTTT-3′ and 5′-CAGACGTATGTCCTGAGCCAT-3′; mouse IL-1β, 5′-TGCCACCTTTTGACAGTGATG-3′ and 5′-TGATGTGCTGCTGCGAGATT-3′; mouse β-actin, 5′-CTGAGAGGGAAATCGTGCGT-3′ and 5′-CCACAGGATTCCATACCCAAGA-3′.

### Statistical analysis

Results are reported as means±SE. Multiple groups were compared by one-way analysis of variance (ANOVA) followed by post-testing with LSD**'**s multiple comparison of means. Statistical significance was set at P<0.05. Pearson's correlation coefficients were calculated to test for associations.

## Results

### Body weight

**Figure 1.**
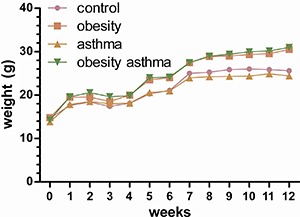
Body weight increase in mice of all groups. The mice fed a high-fat diet gained weight more rapidly than mice fed a normal diet. The final body weight of high-fat diet groups (obesity and obesity asthma) was significantly higher than animals of normal-diet groups (control and asthma) (P<0.05, ANOVA).

Body weight of mice on a high fat diet increased more rapidly than of mice on a normal diet. The final body weight of the high fat diet mice was significantly higher than the lean mice at 12 weeks (Figure 1).

### Airway hyper-responsiveness of mice

**Figure 2.**
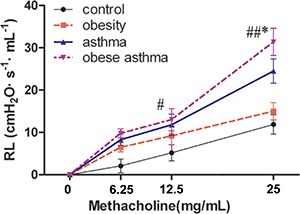
Airway hyper-responsiveness test results. Airways resistance to increasing concentrations of methacholine was determined by the double-chambered whole body plethysmograph and reported as lung resistance (RL, cmH_2_O·s^-1^·mL^-1^). ^#^P<0.05, ^# #^P<0.01, compared with the normal control group; *P<0.05, compared with the asthma group. Data are reported as means±SE (n=5 per group, ANOVA).

Airway responsiveness was gradually increased after OVA immunization in mice after methacholine inhalation compared with the control group. Both the obese group and the asthma group mice showed an increase of airway responsiveness. In obesity with asthma mice, the RL was significantly increased at each concentration of methacholine compared to normal group. Noticeably, at high concentration (25 mg/mL methacholine), airway hyperreactivity of obese asthma mice showed a significant increase compared to asthma mice (P<0.05; Figure 2).

### Cell counts in BALF

**Figure 3.**
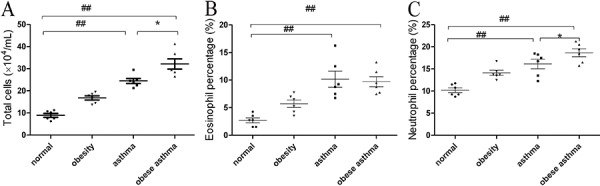
Bronchoalveolar lavage fluid cell counts. *A*, Total inflammatory cell count. *B*, Mean percentage of eosinophils. *C*, Mean percentage of neutrophils. Data are reported as means±SE (n=5/group). ^# #^P<0.01, compared with the normal control group; *P<0.05, compared with the asthma group (ANOVA).

In asthma and obese asthma groups, a significant increase in the number of total inflammatory cells in the BALF was found compared with normal control group (P<0.01). Additionally, the two groups showed a steep rise of the mean percentage of eosinophils and neutrophils in the BALF (P<0.01; Figure 3).

### Histological changes of lung tissues

**Figure 4.**
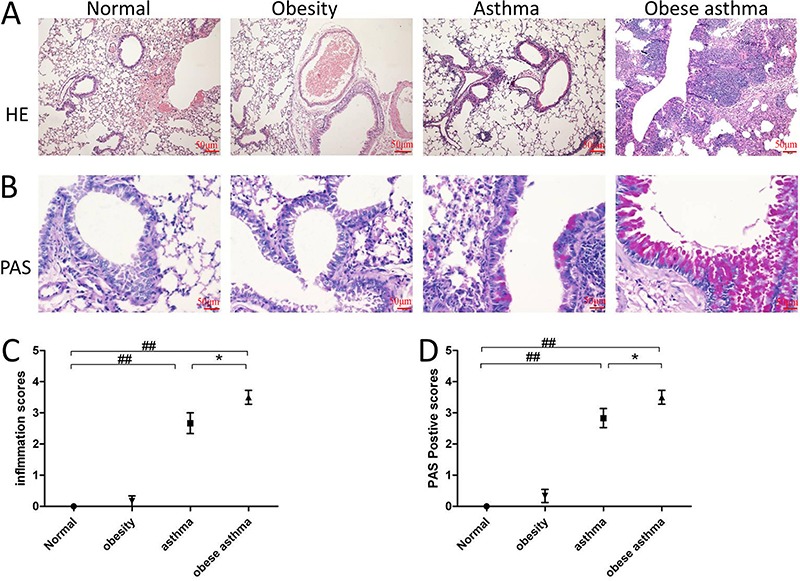
Histologic findings with hematoxylin and eosin (HE) (*A*) and periodic acid-Schiff (PAS) (*B*) staining of lung tissues. *C*, Inflammation scores. *D*, PAS-positive scores. Original magnification: ×400. Data are reported as means±SE (n=5/group). ^# #^P<0.01, compared with the normal control group; *P<0.05, compared with the asthma model group (ANOVA).

Lung histological examination showed structurally normal tissue in control mice. In contrast, mice from the asthma and obese asthma groups exhibited remarkable inflammatory changes with inflammatory cell infiltration and goblet cell hyperplasia. Notably, inflammatory cell infiltration and goblet cell hyperplasia, and mucus hypersecretion in obese asthma mice were more obvious than the asthma mice. (Figure 4A and B). The inflammation scores and mucus scores in all groups are shown in Figure 4C and D.

### Vitamin D3 levels in serum

**Figure 5.**
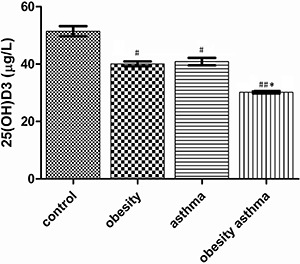
Vitamin D3 (25(OH)D3) levels in serum measured by ELISA. Data are reported as means±SE (n=5/group). ^#^P<0.05, ^# #^P<0.01, significant difference compared with the normal control group; *P<0.05, significant difference compared with the asthma model group (ANOVA).

Vitamin D3 (25(OH)D3) levels (the unit was defined as μg/L) in the obesity and asthma groups (40.086±1.84; 40.876±2.85 μg/L) were decreased compared with the normal control group (51.438±3.97 μg/L; P<0.05). In the obese asthma group, 25(OH)D3 levels (30.196±0.94 μg/L) were significantly reduced compared with the normal control group (P<0.01). The 25(OH)D3 levels in the obese asthma group were the lowest, with a significant difference compared with the obesity and asthma groups. (Figure 5).

### Cytokine concentrations

**Figure 6.**
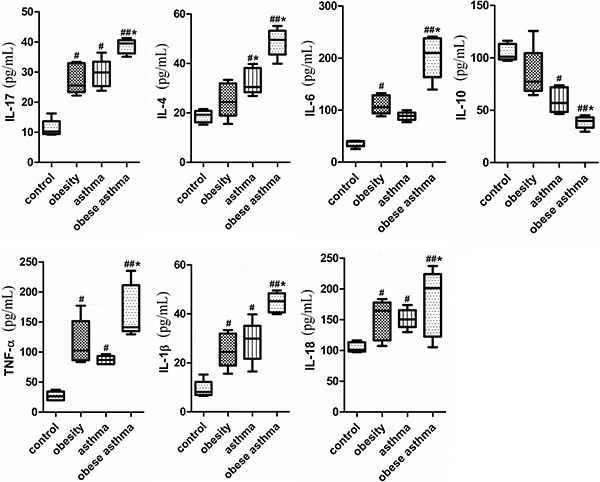
Cytokine levels in bronchoalveolar lavage fluid. Data are reported as means±SE (n=5/group). ^#^P<0.05, ^##^P<0.01, compared with the normal control group; *P<0.05, compared with the asthma group (ANOVA).

The asthma and obesity groups showed an increase in cytokine of IL-17A, IL-4, IL-6, TNF, IL-1β and IL-18 in the BALF compared with normal controls. Furthermore, in the obese asthma group, these pro-inflammatory cytokines were the highest among the 3 groups (Figure 6).

### IL-1β mRNA and NLRP3 mRNA expression

**Figure 7.**
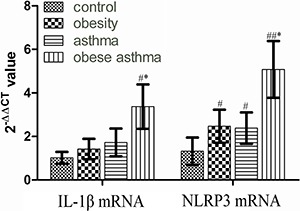
IL-1β mRNA and NLRP3 mRNA expressions in lung examined with qRT-PCR. Data are reported as means±SD (n=5/group). ^#^P<0.05, ^# #^P< 0.01, compared with the normal control group; *P<0.05, compared with the asthma group (ANOVA).

The expression levels of IL-1β mRNA and NLRP3 mRNA in lung tissue of the obesity and asthma groups were increased. In the obese asthma group, IL-1β expression levels were largely increased compared with the normal control group (P<0.05). Moreover, in obese asthma mice, IL-1β mRNA and NLRP3 mRNA expression were significantly increased compared with the asthma and obesity groups (P<0.05; Figure 7).

### Correlation between vitamin D3 and body weight of mice, asthma outcomes, IL-1β and IL-17 levels

**Figure 8.**
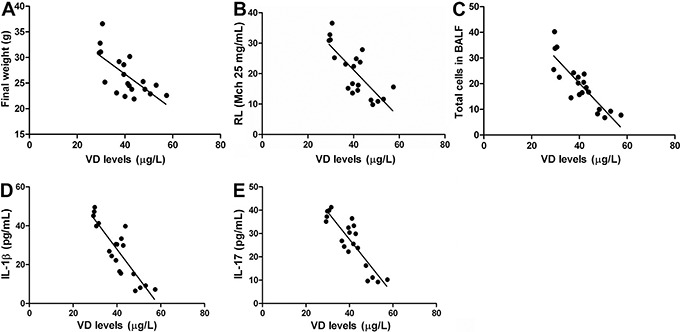
Correlation between vitamin D3 (VD) with (A) final body weight of mice (r^2^=0.4671, P<0.05), (*B*) lung resistance (RL) at 25 mg/mL of methacholine (Mch) (r^2^=0.5905, P<0.05), (*C*) total inflammatory cells in bronchoalveolar lavage fluid (BALF) (r^2^=0.7507, P<0.05) and IL-1β (*D*) and IL-17 (*E*) concentrations in BALF (r^2^=0.7498, P<0.05; r^2^=0.7811, P<0.05).

Vitamin D levels correlated negatively with the final body weight of mice (r^2^=0.4671, P<0.05), with airway resistance at 25 mg/mL methacholine concentration (r^2^=0.5905, P<0.05), with total inflammatory cells in BALF (r^2^=0.7507, P<0.05), and with IL-1β and IL-17 concentration in BALF (r^2^=0.7498, P<0.05; r^2^=0.7811, P<0.05; Figure 8 A-E).

## Discussion

While much epidemiological evidence indicates an obvious link between asthma and obesity, the specific mechanism of the association remains unknown. It is clear that obesity increases the prevalence of asthma and severity of symptoms, and lessens the efficacy of treatment. In view of this, obesity-associated asthma has been listed as a new asthma phenotype with different features compared to other forms of asthma. Of note, obesity is a low-grade, chronic inflammation state. Also, asthma is a chronic inflammatory disease of the lungs. However, there is little evidence on how obesity contributes to lung inflammation and causes the failure of therapies for asthma. Thus, the exact pathogenesis of obesity in asthma needs further elucidation.

Vitamin D has significant effect on immune function in both innate immunity and adaptive immunity. Experimental studies have shown that vitamin D inhibits proliferation of CD4+T cells (5). Other studies reported that vitamin D reduces the production of cytokine IL-17 and IFN-γ (6,7). As for asthma, experimental findings suggest that vitamin D has beneficial effects. Vitamin D insufficiency (a serum 25(OH)D3 level <30 ng/mL) has been associated with asthma morbidity. Using data from the National Health and Nutrition Examination Survey from 2001 to 2010, Han YY et.al (8) revealed that vitamin D insufficiency was associated with current asthma and current wheeze in children, as well as with current wheeze in adults. Observational studies and a small clinical trial suggest that vitamin D protects against asthma exacerbations. Kreindler et al. (9) revealed that vitamin D increased TGF-β-positive Tregs and lowered Th2 cytokine levels. Schedel et al. (10) showed that addition of 1,25D3, the active form of vitamin D3, during CD8+T-cell differentiation prevents IL-4-induced conversion to IL-13-producers. On the other hand, low levels of circulating vitamin D3 correlate with high adiposity (11). Obesity-associated vitamin D insufficiency is likely due to the decreased bioavailability of vitamin D3 from cutaneous and dietary sources because of its deposition in body fat compartments (12).

Scott et al. (13) found that obese asthma exhibits a significant neutrophilic airway inflammation. Airway neutrophilia has been reported to be a typical feature of severe asthma (14). NLRP3 inflammasome is upregulated in neutrophilic asthma and may regulate the inflammation process through production of IL-1β (15). Recently, Kim et al. (16) also reported that NLRP3 inflammasome activation play key roles in severe, steroid-resistant asthma.

In the present study, we investigated vitamin D3 levels, airway hyper-responsiveness, cytokine levels, and NLRP3 and IL-1β mRNA expressions by developing a mouse model of obese asthma via OVA-challenge and high-fat diet. The non-invasive methacholine bronchial provocation test by a whole-body plethysmography revealed that obese asthma mice developed significantly increased airway responsiveness. In addition, we observed significantly increased IL-17, IL-4, IL-1β and IL-18 etc. in BALF. Furthermore, obese asthma mice exhibited significantly lower vitamin D levels in serum and significantly higher NLRP3 and IL-1β mRNA expression levels in lung tissue. Moreover, serum vitamin D levels were inversely correlated with airway resistance, total inflammatory cells, and IL-1β and IL-17 concentrations in BALF, suggesting that vitamin D3 level is a potential predictor for obese asthma. Lower vitamin D3 levels may influence proinflammatory cytokines and NLRP3 inflammasome activity in obese asthma. It appears that NLRP3 inflammasome is involved in the interaction of vitamin D3 in obesity with asthma. This finding may facilitate the development of novel therapeutic targets for obesity patients with asthma. Nevertheless, further studies are needed to determine if vitamin D supplementation can improve outcomes in obesity patients with asthma and the exact role of vitamin D in regulating NLRP3 inflammasome activity.

## References

[1] Koebnick C, Fischer H, Daley MF, Ferrara A, Horberg MA, Waitzfelder B (2016). Interacting effects of obesity, race, ethnicity and sex on the incidence and control of adult-onset asthma. Allergy Asthma Clin Immunol.

[2] Chen YP, Zhang JH, Li CQ, Sun QX, Jiang XH (2015). Obesity enhances Th2 inflammatory response via natural killer T cells in a murine model of allergic asthma. Int J Clin Exp Med.

[3] Zhang J, Li C, Guo S (2012). Effects of inhaled inactivated Mycobacterium phlei on airway inflammation in mouse asthmatic models. J Aerosol Med Pulm Drug Deliv.

[4] Li C, Jiang X, Luo M, Feng G, Sun Q, Chen Y (2016). Mycobacterium vaccae nebulization can protect against asthma in Balb/c mice by regulating Th9 expression. PLoS One.

[5] Mahon BD, Wittke A, Weaver V, Cantorna MT (2003). The targets of vitamin D depend on the differentiation and activation status of CD4 positive T cells. J Cell Biochem.

[6] Reichel H, Koeffler HP, Tobler A, Norman AW (1987). 1 alpha,25-Dihydroxyvitamin D3 inhibits gamma-interferon synthesis by normal human peripheral blood lymphocytes. Proc Natl Acad Sci U S A.

[7] Tang J, Zhou R, Luger D, Zhu W, Silver PB, Grajewski RS (2009). Calcitriol suppresses antiretinal autoimmunity through inhibitory effects on the Th17 effector response. J Immunol.

[8] Han YY, Forno E, Celedon JC (2017). Vitamin D insufficiency and asthma in a US Nationwide Study. J Allergy Clin Immunol Pract.

[9] Kreindler JL, Steele C, Nguyen N, Chan YR, Pilewski JM, Alcorn JF (2010). Vitamin D3 attenuates Th2 responses to Aspergillus fumigatus mounted by CD4+ T cells from cystic fibrosis patients with allergic bronchopulmonary aspergillosis. J Clin Invest.

[10] Schedel M, Jia Y, Michel S, Takeda K, Domenico J, Joetham A (2016). 1,25D3 prevents CD8(+)Tc2 skewing and asthma development through VDR binding changes to the Cyp11a1 promoter. Nat Commun.

[11] Trinko JR, Land BB, Solecki WB, Wickham RJ, Tellez LA, Maldonado-Aviles J (2016). Vitamin D3: A role in dopamine circuit regulation, diet-induced obesity, and drug consumption. eNeuro.

[12] Wortsman J, Matsuoka LY, Chen TC, Lu Z, Holick MF (2000). Decreased bioavailability of vitamin D in obesity. Am J Clin Nutr.

[13] Scott HA, Gibson PG, Garg ML, Wood LG (2011). Airway inflammation is augmented by obesity and fatty acids in asthma. Eur Respir J.

[14] Ordonez CL, Shaughnessy TE, Matthay MA, Fahy JV (2000). Increased neutrophil numbers and IL-8 levels in airway secretions in acute severe asthma: Clinical and biologic significance. Am J Respir Crit Care Med.

[15] Simpson JL, Phipps S, Baines KJ, Oreo KM, Gunawardhana L, Gibson PG (2014). Elevated expression of the NLRP3 inflammasome in neutrophilic asthma. Eur Respir J.

[16] Kim RY, Pinkerton JW, Essilfie AT, Robertson AA, Baines KJ, Brown AC (2017). Role for NLRP3 inflammasome-mediated, IL-1beta-dependent responses in severe, steroid-resistant asthma. Am J Respir Crit Care Med.

